# *CYP17 *gene polymorphism in relation to breast cancer risk: a case-control study

**DOI:** 10.1186/bcr1319

**Published:** 2005-09-14

**Authors:** Kristjana Einarsdóttir, Tove Rylander-Rudqvist, Keith Humphreys, Susanne Ahlberg, Gudrun Jonasdottir, Elisabete Weiderpass, Kee Seng Chia, Magnus Ingelman-Sundberg, Ingemar Persson, Jianjun Liu, Per Hall, Sara Wedrén

**Affiliations:** 1 Department of Medical Epidemiology and Biostatistics, Karolinska Institutet, Stockholm, Sweden; 2 Population Genetics, Genome Institute of Singapore, Singapore; 3 Division of Nutritional Epidemiology, Institute of Environmental Medicine, Karolinska Institutet, Stockholm, Sweden; 4 Division of Molecular Toxicology, Institute of Environmental Medicine, Karolinska Institutet, Stockholm, Sweden; 5 Cancer Registry of Norway, Montebello, Oslo, Norway; 6 Centre for Molecular Epidemiology, Department of Community, Occupational and Family Medicine, National University of Singapore, Singapore; 7 Swedish Medical Products Agency, Uppsala, Sweden

## Abstract

**Introduction:**

The c.1-34T>C 5' promoter region polymorphism in cytochrome P450c17 (CYP17), a key enzyme in the biosynthesis of estrogen, has been associated with breast cancer risk, but most previous studies have been relatively small.

**Methods:**

We genotyped 1,544 incident cases of primary breast cancer and 1,502 population controls, all postmenopausal Swedish women, for the *CYP17 *c.1-34T>C polymorphism and calculated odds ratios (ORs) and 95% confidence intervals (CIs) from logistic regression models.

**Results:**

No overall association was found between *CYP17 *c.1-34T>C and breast cancer risk, OR 1.0 (95% CI 0.8–1.3) for the A2/A2 (CC) carriers compared to the A1/A1 (TT) carriers, regardless of histopathology. We detected an interaction between *CYP17 *c.1-34T>C and age at menarche (P = 0.026) but regarded that as a chance finding as no dose-response pattern was evident. Other breast cancer risk factors, including menopausal hormone use and diabetes mellitus, did not modify the overall results.

**Conclusion:**

It is unlikely that *CYP17 *c.1-34T>C has a role in breast cancer etiology, overall or in combination with established non-genetic breast cancer risk factors.

## Introduction

Estrogen is a central factor in the etiology of breast cancer [1]. One of the key enzymes in the synthesis of sex hormones, such as estrogens and androgens, is cytochrome P450c17 (CYP17). CYP17 catalyses the 17α-hydroxylation of pregnenolone and progesterone, and these intermediates are then converted to dehydroepiandosterone and androstenedione by the 17,20-lyase activity of the enzyme [2,3].

A single T (A1 allele) to C (A2 allele) base change in the 5' promoter region of *CYP17 *(c.1-34T>C) has been suggested to create an additional binding site for the transcription factor Sp1 [4]. This could theoretically lead to increased levels of the enzyme, but use of the extra binding site has not been confirmed experimentally [5]. Three groups have reported an association of the A2 allele with increased breast cancer risk [6-8] but others have failed to replicate those findings [5,9-21]. A recent meta-analysis of 15 case-control studies did not find any overall association [22] but this analysis was criticized by Feigelson and colleagues [23], who found a borderline significant association between the *CYP17 *polymorphism and advanced breast cancer.

In a population-based study about causes of postmenopausal breast cancer, which included 1,544 cases and 1,502 controls, we evaluated the association between *CYP17 *c.1-34T>C and breast cancer risk among Swedish women. We also explored potential interaction between genotype and menopausal hormone use and diabetes mellitus as well as age at menarche, age at menopause, age at first birth, parity and body mass index.

## Materials and methods

### Founding study

This nation-wide case-control study included all Swedish women, 50 to 74 years of age and resident in Sweden between October 1993 and March 1995. During that period, all incident cases of primary invasive breast cancer were identified at diagnosis through the six regional cancer registries in Sweden, to which reporting of all malignant tumors is mandatory. Controls that matched the cases in 5-year age strata were randomly selected from the Swedish Registry of Total Population. Of the eligible cases and controls, 3,345 (84%) and 3,454 (82%), respectively, participated in the study by providing detailed information via questionnaires about menopausal hormone use, reproductive history, and other lifestyle factors. Results from the founding study have been published [24-28].

### Collection of biological samples

Only postmenopausal women with a first primary breast cancer without any previous malignancy (except carcinoma *in situ *of the cervix or non-melanoma skin cancer) were eligible for the present study. Premenopausal women and those with a previous malignancy were excluded. From the eligible participants, we randomly selected 1,500 cases and 1,500 age-frequency (in 5-year groups) matched controls. We were interested in exploring the interaction between *CYP17 *c.1-34T>C and menopausal hormones and diabetes mellitus as both are breast cancer risk factors, possibly acting through increased levels of exogenous and endogenous estrogens, respectively. Thus, in order to increase statistical power in the subgroup analyses, we further selected all remaining eligible women (191 cases and 108 controls) that had used menopausal hormones (estrogen only or any combination of estrogen and progestin) for at least 4 years and all women (110 cases and 104 controls) with self-reported diabetes mellitus. In addition, 345 eligible controls from the founding study, originally selected for a parallel endometrial cancer study but who fulfilled the inclusion criteria to the breast cancer study, were added to our control sample. In total, we selected 1,801 cases and 2,057 controls.

Paraffin-embedded, non-cancerous tissue samples were collected for deceased breast cancer cases as well as for those breast cancer cases that declined to donate blood but consented to our use of the tissue. We obtained blood samples or archived tissue samples for 1,322 and 247 breast cancer patients, respectively, and blood samples from 1,524 controls. Population-based participation rates (taking into account those that did not participate in the founding study but could have been selected) for cases and controls were 73% and 61%, respectively.

We isolated DNA from 3 ml of whole blood using a Wizard Genomic DNA Purification Kit (Promega, Madison, WI, USA) according to the manufacturer's instructions. From non-malignant cells in paraffin-embedded tissue, we extracted DNA using a standard phenol/chloroform/isoamyl alcohol protocol [29]. The study was approved by the five Swedish Institutional Review Boards and was performed in compliance with the Helsinki Declaration.

### Genetic analyses

We used two methods for *CYP17 *c.1-34T>C (rs743572) genotyping: Multiplex fluorescent solid-phase minisequencing [30]; and dynamic allele specific hybridization (DASH) [31]. Results from the two methods were validated with PCR-RFLP [32]. Twenty-four percent of the samples were analysed with both minisequencing and DASH, and the genotypes obtained were identical. Primer and probe sequences and PCR reaction conditions are given in Additional file 1. The PCR reactions were performed on a Perkin Elmer GeneAmp 9700 system (PerkinElmer Life And Analytical Sciences, Inc., Boston, MA, USA).

### Statistical analyses

Odds ratios (ORs) and 95% confidence intervals (CIs) were calculated using conditional logistic regression models with the A1/A1 genotype as the reference group. We conditioned the models on age (due to matching) and on duration of menopausal hormone use and self-reported diabetes mellitus (due to our over-sampling scheme). The conditioning variables were thus age (5-year age groups), menopausal estrogen only use (never, <4 years or ≥4 years), menopausal estrogen and progestin use (never, <4 years or ≥4 years) and self-reported diabetes mellitus (yes or no). We chose to condition because in the presence of interaction between any of the selection variables and *CYP17 *genotype, estimates of the main effect of *CYP17 *could be biased. We did not expect confounding by other known breast cancer risk factors as they most likely would be intermediates between the genetic variant and breast cancer. We nevertheless included important breast cancer risk factors as co-variates in the models and assessed changes in the estimates of association between *CYP17 *genotype and breast cancer to investigate if there were any indications of such interrelations. Breast cancer risk factors other than menopausal hormone use that were analyzed for gene-environment interaction were: Age at menarche (≤2 years, >12–14 years or >14 years) and age at menopause (<49 years, 49–52 years or >52 years) (where 1^st ^category included the 1^st ^quartile of the data, 2^nd ^category the 2^nd ^and 3^rd ^quartiles, and 3^rd ^category the 4^th ^quartile); age at first birth (≤24 years, 25–29 years or ≥30 years); parity (nulliparous, 1 child, 2 children or >2 children); and body mass index one year prior to diagnosis (<25, 25 to <28, or ≥28). The last three were categorized according to cut-offs that in previous studies have been shown to be informative with regard to the variables' influence on breast cancer risk. The likelihood ratio test and the Wald test statistic were used to test for interaction. We performed all analyses using the SAS system (release 8.01; SAS Institute Inc., Cary, NC, USA).

## Results

We obtained *CYP17 *c.1-34T>C genotypes from 1,544 breast cancer cases and 1,502 controls. Among the controls, genotype frequencies were similar to previously published frequencies in Caucasian populations [5,9-11,20,21] and did not deviate from Hardy-Weinberg Equilibrium (P = 0.927). Genotype frequencies among the cases did not differ between blood and tissue samples (P = 0.697).

We reproduced previously established breast cancer risk factor associations in this study population [24,25,33] (Table 1, first column). The one exception was age at menarche, which did not appear to be associated with overall breast cancer risk in this study [25].

The *CYP17 *c.1-34T>C was not associated with other breast cancer risk factors among the randomly selected controls (Table 2).

We found no overall association between *CYP17 *c.1-34T>C and breast cancer risk, regardless of histopathology (Table 3). This negative result was not modified by menopausal hormone use or diabetes mellitus (Table 3). We also did not detect any association when we considered stage 1 (n = 389) and stage 2 or more advanced (n = 591) breast cancers separately (data not shown). None of these findings were altered by restricting the sample set to the randomly selected cases and controls or by including other breast cancer risk factors as co-variates in the logistic regression models.

In exploratory analyses, the A2/A2 genotype, compared to A1/A1, was associated with an increased breast cancer risk in women with age at menarche of 12 years or younger (OR 1.9, 95% CI 1.1–3.0; P for interaction = 0.026; Table 1). Furthermore, the A2 allele in carriers conferred a decreased risk in women with menopause before 49 years of age; OR 0.7 (95% CI 0.5–1.0) for heterozygotes and OR 0.7 (95% CI 0.4–1.1) for homozygotes) compared to A1/A1. There was, however, no dose-response pattern in these findings and we therefore regarded them as being due to chance. Age at first birth, parity or body mass index did not seem to modify the non-association between *CYP17 *c.1-34T>C genotype and breast cancer risk (Table 1). All estimates remained unaffected after we restricted the analyses to the randomly selected cases and controls.

## Discussion

*CYP17 *c.1-34T>C was not associated with breast cancer risk overall, a result that did not seem to be modified by menopausal hormone use or diabetes mellitus. Incidentally, we detected a statistically significant interaction between *CYP17 *c.1-34T>C and age at menarche but regarded the finding as a chance result as no dose-response pattern was evident.

Our population-based study is one of the largest published to date, investigating the possible influence of *CYP17 *variation on breast cancer risk. Retrieval of extensive information on reproductive and lifestyle factors enabled us to test for gene-environment interactions. Misclassification of genotypes should be minimal as is indicated by the identical results from replicate genotyping with two different methods, all blinded to case-control status. Any genotype misclassification despite these measures is unlikely to have been differential and would consequently only bias our estimates towards the null.

Because pre- and postmenopausal breast cancer is conceived to have differing causal mechanisms, these two types of cancer are often investigated separately. We were interested in potential interaction between *CYP17 *genotype and menopausal hormone treatment, which motivated our restricting the study population to postmenopausal women. Although it would be interesting to know also if *CYP17 *genotype influences the risk of premenopausal breast cancer, had we included both pre- and postmenopausal women, the study size would have had to be increased to have the same power to address our main hypotheses. We were also interested in exploring the interaction with diabetes mellitus as insulin resistance can lead to increased levels of plasma insulin, lower levels of plasma sex hormone binding globulin and, consequently, increased levels of free estrogen [34]. We thus over-sampled menopausal hormone users and diabetics in the design phase of the study so as to increase statistical precision in subgroup analyses. We explored only the effect of *CYP17 *c.1-34T>C on breast cancer risk in this article, but 13 variants in 7 genes were genotyped in the current phase of the study. Results from three of the studied genes in relation to breast cancer risk have previously been published [35-37].

The lack of association between *CYP17 *c.1-34T>C and overall breast cancer risk in our study is in line with results from 14 previous studies – where 10 included only Caucasian women – and a recent meta-analysis [5,9-22]. In contrast to Feigelson and colleagues [8], we did not find any association between this polymorphism and advanced breast cancer. Two groups have reported an association between *CYP17 *genotype and breast cancer risk in postmenopausal women [6,7]. All three studies were performed in non-Caucasian populations.

A possible mechanism for the *CYP17 *c.1-34T>C polymorphism to influence breast cancer risk is through increased biosynthesis of and, thereby, increased levels of circulating estrogen. Contrary to the predicted effect of the A2 allele, one group found decreased *CYP17 *mRNA levels in A2 carriers [38]. Studies regarding association of *CYP17 *c.1-34T>C and circulating hormone levels as well as markers of hormonal status (i.e. age at menarche or menopausal hormone use) have recently been reviewed [39]. Increased estradiol levels have been associated with the A2 allele in premenopausal women [6,32] as well as in postmenopausal women [9,40], but three groups did not report any significant changes in hormone levels by genotype, two in postmenopausal women [17,41] and one in premenopausal women [42]. Results from seven studies have indicated a moderate association between the A2 allele and earlier menarche [8,11,15,18,43-45], an association that was not detected in our study, nor in five others [9,12,21,46,47]. Furthermore, previous investigators have posited that *CYP17 *genotype may be associated with use of menopausal hormones, an important risk factor for breast cancer, but results have been inconsistent [11,12,21,48-50]. We found no such association in our data.

We identified an interaction with age at menarche, but considered it unlikely that the A2/A2 genotype would increase breast cancer risk in women with age at menarche less than 13 years without also moderately increasing risk in women with age at menarche between 12 and 14 years. Instead, the A2/A2 genotype decreased risk in the latter group, which is difficult to explain biologically. A similar pattern was seen for the interaction with age at menopause. To our knowledge, other groups have not reported comparable findings and we therefore believe the results to be caused by chance alone.

## Conclusion

*CYP17 *c.1-34T>C does not appear to have any major influence on breast cancer risk. Despite this negative finding, it would be prudent to investigate common variants covering the entire *CYP17 *gene before concluding that this gene has no role in breast cancer etiology.

## Abbreviations

CI = confidence interval; CYP17 = cytochrome P450c17; DASH = dynamic allele-specific hybridization; OR = odds ratio; PCR = polymerase chain reaction; RFLP = restriction fragment length polymorphism.

## Competing interests

The authors declare that they have no competing interests.

## Authors' contributions

Kristjana Einarsdóttir wrote the article and performed the statistical analyses. Keith Humphreys and Gudrun Jonasdottir advised on the statistical methods. Tove Rylander-Rudqvist and Susanne Ahlberg performed the genetic analyses. Magnus Ingelman-Sundberg and Ingemar Persson conceived and designed the study. Elisabete Weiderpass oversaw the project progress and took part in interpretation and writing the article. Jianjun Liu and Kee Seng Chia critically revised the manuscript. Per Hall was involved in drafting the article, assisted in interpretation of data, and gave final approval of the version to be published. Sara Wedrén contributed to conception and design of the project, coordinated sample collection, was involved in writing the article, assisted in analysis and interpretation of data and gave final approval for publication. All authors read and approved the final manuscript.

## Supplementary Material

Additional File 1 Microsoft Word document detailing the PCR reaction conditions as well as primer and probe sequences used to perform the minisequencing and DASH methods. Click here for file

## Figures and Tables

**Table 1:** Breast cancer risk in relation to *CYP17 *genotype, stratified by breast cancer risk factors

	All genotypes		*CYP17 *genotype						P for interaction
			A1/A1		A1/A2		A2/A2		

	Cases/controls^a^	OR^b ^(CI)	Cases/controls^a^	OR^b ^(CI)	Cases/controls^a^	OR^b ^(CI)	Cases/controls^a^	OR^b ^(CI)	

Age at menarche (years)									
≤12	314/262	1 (reference)	102/96	1 (reference)	138/128	1.1 (0.7–1.5)	74/38	1.9 (1.1–3.0)	
>12–14	749/702	0.9 (0.7–1.1)	283/251	1.1 (0.8–1.5)	360/325	1.1 (0.8–1.5)	106/126	0.8 (0.6–1.0)	
>14	300/258	1.0 (0.8–1.2)	107/94	1.1 (0.7–1.6)	152/126	1.2 (0.8–1.7)	41/38	1.1 (0.6–1.8)	0.026
Age at menopause (years)									
<49	330/347	1 (reference)	145/123	1 (reference)	141/165	0.7 (0.5–1.0)	44/59	0.7 (0.4–1.1)	
49–52	802/656	1.3 (1.1–1.6)	282/243	1.0 (0.7–1.3)	380/316	1.0 (0.8–1.4)	140/97	1.2 (0.9–1.8)	
>52	357/323	1.2 (1.0–1.5)	119/117	1.0 (0.6–1.3)	184/150	1.1 (0.8–1.6)	54/56	0.8 (0.5–1.3)	0.062
Age at first birth (years)									
≤24	616/639	1 (reference)	225/236	1 (reference)	310/315	1.1 (0.8–1.3)	81/88	1.0 (0.7–1.4)	
25–29	414/388	1.1 (0.9–1.4)	145/144	1.1 (0.8–1.5)	187/171	1.2 (0.9–1.6)	82/73	1.2 (0.8–1.7)	
≥30	242/187	1.4 (1.1–1.7)	100/67	1.6 (1.1–2.3)	107/89	1.3 (0.9–1.8)	35/31	1.1 (0.7–1.9)	0.752
Parity									
Nulliparous	224/124	1 (reference)	79/41	1 (reference)	105/63	0.9 (0.5–1.5)	40/20	1.2 (0.6–2.3)	
1 child	320/240	0.7 (0.5–0.9)	113/77	0.8 (0.5–1.2)	168/130	0.7 (0.4–1.0)	39/33	0.6 (0.3–1.1)	
2 children	569/515	0.6 (0.4–0.7)	215/195	0.6 (0.4–0.9)	254/238	0.5 (0.4–0.8)	100/82	0.6 (0.4–1.0)	
>2 children	386/459	0.5 (0.4–0.6)	143/175	0.4 (0.3–0.7)	184/207	0.5 (0.3–0.7)	59/77	0.4 (0.2–0.6)	0.792
Body mass index (kg/m^2^)									
<25	716/669	1 (reference)	263/230	1 (reference)	337/329	0.9 (0.7–1.2)	116/110	0.9 (0.7–1.3)	
25 to <28	371/377	1.0 (0.8–1.1)	141/137	0.9 (0.7–1.2)	170/181	0.9 (0.7–1.2)	60/59	0.9 (0.6–1.4)	
≥28	403/278	145/116	1.2 (0.9–1.6)	198/121	1.4 (1.1–1.9)	60/41	1.4 (0.9–2.2)	0.657	

^a^Including only cases and controls with complete information on menopausal hormones and diabetes mellitus. ^b^Analyses were conditioned on age (5-year age groups), menopausal estrogen only use (never, <4 years or ≥4 years), use of estrogen in combination with progestin (never, <4 years or ≥4 years) and diabetes mellitus (yes or no). CI, confidence interval; OR, odds ratio.

**Table 2:** Descriptive characteristics of postmenopausal breast cancer cases and controls by *CYP17 *c.1-34T>C genotype

	*CYP17 *genotype								
	A1/A1			A1/A2			A2/A2		

Characteristic	Cases/controls^a^	Cases	Controls	Cases/controls^a^	Cases	Controls	Cases/controls^a^	Cases	Controls

Genotype frequencies	550/488	36.7	36.5	711/638	47.4	47.7	238/212	15.9	15.8
Age (years)^b^	550/488	63.3 ± 6.5	63.0 ± 6.5	711/638	63.4 ± 6.5	62.9 ± 6.4	238/212	62.8 ± 6.6	63.2 ± 6.1
Age at menarche (years)^b^	492/441	13.5 ± 1.4	13.5 ± 1.4	650/579	13.6 ± 1.4	13.5 ± 1.4	221/202	13.2 ± 1.5	13.5 ± 1.3
Age at menopause (years)^b^	546/483	49.9 ± 3.8	50.1 ± 4.1	705/631	50.7 ± 3.2	50.0 ± 3.8	238/212	50.6 ± 3.2	50.2 ± 4.2
Age at first birth (years)^b^	470/447	25.4 ± 5.0	24.7 ± 4.6	604/575	25.1 ± 5.0	24.7 ± 4.7	198/192	25.6 ± 4.5	25.2 ± 4.9
Parity^b^	550/488	1.9 ± 1.2	2.3 ± 1.4	711/638	1.8 ± 1.2	2.1 ± 1.2	238/212	1.8 ± 1.2	2.2 ± 1.3
Body mass index (kg/m^2^)^b^	549/483	25.8 ± 4.1	25.8 ± 4.4	705/631	25.9 ± 4.2	25.2 ± 3.8	236/210	25.7 ± 4.4	25.3 ± 4.0
Duration of menopausal hormone use (years)^c^									
0	390/370	70.9	75.8	469/473	66.0	74.1	165/154	69.3	72.6
<4	59/50	10.7	10.3	96/74	13.5	11.6	26/25	10.9	11.8
≥4^d^	101/68	18.4	13.9	146/91	20.5	14.3	47/33	19.8	15.6
Self-reported diabetes mellitus^c,d^	550/488	8.73	8.20	711/638	9.42	7.21	238/212	8.82	8.49

^a^Including only cases and controls with complete information on menopausal hormones and diabetes mellitus. ^b^Means ± standard deviation. ^c^Percentages. ^d^Long term users of menopausal hormones and women with diabetes mellitus were over-sampled.

**Table 3:** Breast cancer risk in relation to *CYP17 *genotype, stratified by histopathological type, menopausal hormones or diabetes mellitus

	*CYP17 *genotype						P for interaction
	A1/A1		A1/A2		A2/A2		

	Cases/controls^a^	OR^b^(CI)	Cases/controls^a^	OR^b^(CI)	Cases/controls^a^	OR^b^(CI)	

All cancers	550/488	1 (reference)	711/638	1.0 (0.9–1.2)	238/212	1.0 (0.8–1.3)	
Ductal cancers	420/488	1 (reference)	510/638	1.0 (0.8–1.1)	180/212	1.0 (0.8–1.3)	
Lobular cancers	56/488	1 (reference)	90/638	1.3 (0.9–1.8)	24/212	1.1 (0.6–1.8)	
Duration of menopausal hormone use(all cancers)							
Never	390/370	1 (reference)	469/473	0.9 (0.8–1.1)	165/154	1.0 (0.8–1.3)	
Any kind							
<4	59/50	1 (reference)	96/74	1.2 (0.7–2.0)	26/25	1.0 (0.5–2.1)	
≥4	101/68	1 (reference)	146/91	1.2 (0.8–1.9)	47/33	1.0 (0.6–1.7)	0.668
Estrogen only							
<4	28/22	1 (reference)	43/33	1.1 (0.5–2.3)	7/11	0.6 (0.2–1.8)	
≥4	39/23	1 (reference)	42/30	1.0 (0.5–2.1)	15/13	0.7 (0.3–1.9)	0.703
Estrogen + progestin							
<4	42/41	1 (reference)	75/66	1.2 (0.7–2.1)	23/21	1.2 (0.6–2.5)	
≥4	64/47	1 (reference)	108/62	1.3 (0.8–2.2)	34/20	1.2 (0.6–2.4)	0.723
Self-reported diabetes mellitus (all cancers)							
No	502/448	1 (reference)	644/592	1.0 (0.8–1.2)	217/194	1.0 (0.8–1.3)	
Yes	48/40	1 (reference)	67/46	1.3 (0.7–2.2)	21/18	1.1 (0.5–2.3)	0.259

^a^Including only cases and controls with complete information on menopausal hormones and diabetes mellitus. ^b^Analyses were conditioned on age (5-year age groups), menopausal estrogen only use (never, <4 years or ≥4 years), use of estrogen in combination with progestin (never, <4 years or ≥4 years) and diabetes mellitus (yes or no). CI, confidence interval; OR, odds ratio.
