# Supplementary material for: CYP17 gene polymorphism in relation to breast cancer risk: a case-control study
Source: Breast Cancer Res. 2005 Sep 14;7(6):R890–6. doi: 10.1186/bcr1319 (PMC1410739; doi:10.1186/bcr1319)
Supplement: Additional File 1 — Microsoft Word document detailing the PCR reaction conditions as well as primer and probe sequences used to perform the minisequencing and DASH methods. [file bcr1319-S1.doc]

**Minisequencing**

The *CYP17* c.1-34T>C was detected by specific extension with single fluorescein labelled (NEN/DuPont, Boston, USA) ddNTPs, of a primer that anneals immediately adjacent to the variable site. To minimize pipetting steps, an AutoLoad kit (Amersham, Pharmacia Biotech, Uppsala, Sweden) where polymerase chain reaction (PCR) products were immobilized on streptavidin-coated comb shaped manifold supports was used. The 25 μl PCR reactions contained approximately 100 ng DNA, 20 mM Tris-HCl pH 8.4, 50 mM KCl, 0.2 mM of each dNTP, 1.8 mM MgCl_2_, 10% DMSO, 0.6 U of Platinum Taq polymerase (Invitrogen, CA), and 0.25 μM of each multiplex PCR primer. The initial denaturation was performed at 95^o^C for 2 min, followed by 35 cycles each consisting of denaturation at 95^o^C for 15 s, annealing at 56^o^C and extension at 72^o^C for 1 min, followed by a final extension at 72^o^C for 7 min. All samples were analyzed on agarose gels to verify that all fragments in the multiplex PCR reaction had been amplified. Six μl biotinylated PCR product was immobilized on streptavidin-coated sequencing combs. The minisequencing procedure was performed as described by Pastinen et al. 1996 [1], with the following modifications; the four minisequencing reaction mixtures contained 26 mM Tris-HCl pH 9.5, 6.5 mM MgCl_2_, 2 μM of each sequencing primer, 0.05 μM of F-ddGTP or 0.25 μM of F-ddATP or 0.5 μM of F-ddCTP or 0.5 μM of F-ddUTP, 0.5 μM of the three other unlabelled ddNTPs and 0.26 units of Thermo Sequenase DNA polymerase (Amersham Pharmacia Biotech). The extended primers where analysed on an ALF DNA-Autosequencer (Amersham, Pharmacia Biotech) and the chromatograms were interpreted by direct visual inspection.

**DASH**

The PCR mix contained 10 ng DNA, 15 mM Tris-HCl pH 8.0, 50 mM KCl, 0.12 μM biotinylated 5´-primer, 0.6 μM 3´-primer (Table 1), 0.2 mM of each dNTP (HPLC purified, Interactiva GmbH, Ulm, Germany), 3.0 mM MgCl_2_, 5% DMSO, 0.6 units of AmpliTaq GOLD polymerase (Applied Biosystems, Foster City, CA) in a total volume of 25 μl. The initial denaturation was performed at 95^o^C for 10 min, followed by 38 cycles each consisting of denaturation at 95^o^C for 15 s and annealing at 60^o^C for 30 s, followed by a final extension at 72^o^C for 4 min. PCR products were checked on 2% low-melting agarose gels. DASH assays were performed as described by Prince et al. 2001 [2] and genotypes were scored from fluorescence curves as described by Howell et al. 1999 [3].

**References**

1. Pastinen T, Partanen J, Syvanen AC: **Multiplex, fluorescent, solid-phase minisequencing for efficient screening of DNA sequence variation.** *Clin Chem* 1996, **42:**1391-7.

2. Prince JA, Feuk L, Howell WM, Jobs M, Emahazion T, Blennow K, Brookes AJ: **Robust and accurate single nucleotide polymorphism genotyping by dynamic allele-specific hybridization (DASH): design criteria and assay validation.** *Genome Res* 2001, **11:**152-62.

3. Howell WM, Jobs M, Gyllensten U, Brookes AJ: **Dynamic allele-specific hybridization. A new method for scoring single nucleotide polymorphisms.** *Nat Biotechnol* 1999, **17:**87-8.

***Table 1. Primers and probes used for genotyping CYP17 c.1-34T>C***

|  |  |  | 5’ – 3’ |
| --- | --- | --- | --- |
| PCR primers | Minisequencing | Forward | AGTCAAGGTGAAGATCAGGG |
|  |  | Reverse | Bio-GCTCTTGGGGTACTTGGCAC |
|  | DASH | Forward | Bio-GAGTTGCCAGAGCTCTTCTAC |
|  |  | Reverse | GGTGCCGGCAGGCAAGATAGA |
|  |  |  |  |
| Probes | Minisequencing | Extension primer | AGAGTTGCCACAGCTCTTCTACTCCAC |
|  | DASH | Wildtype | TAGACAGCAGTGGAGTA |
|  |  | Mutant | TAGACAGCGGTGGAGTA |
